# Supplementary material for: Regulation of Arabidopsis photoreceptor CRY2 by two distinct E3 ubiquitin ligases
Source: Nat Commun. 2021 Apr 12;12:2155. doi: 10.1038/s41467-021-22410-x (PMC8042123; doi:10.1038/s41467-021-22410-x)
Supplement: Supplementary file 1 — Supplementary Information [file 41467_2021_22410_MOESM1_ESM.pdf]

Supplementary information

Regulation of *Arabidopsis* photoreceptor CRY2 by two distinct E3 ubiquitin ligases

Contents

Supplementary Fig. 1 Flowering phenotype analysis of *cry1cry2lrb123* quintuple mutant plants. (for Figure 1)

Supplementary Fig. 2 Rescue of the blue-light hypersensitivity phenotype of *lrb123* by LRB2. (for Figure 1)

Supplementary Fig. 3 CRY2^{P532L} plant is insensitive to blue light. (for Figure 6)

Supplementary Fig. 4 Maps of HEK293T vectors used in this study.

Supplementary Fig. 5 Maps of plant binary vectors used in this study.

Supplementary Table 1 CRY2-IP mass-spectrometric results showing the blue light-dependent association of CRY2/LRB1 and CRY2/LRB2 in *Arabidopsis*. (for Figure 3 and Figure 4)

Supplementary Table 2 Primers used in this study

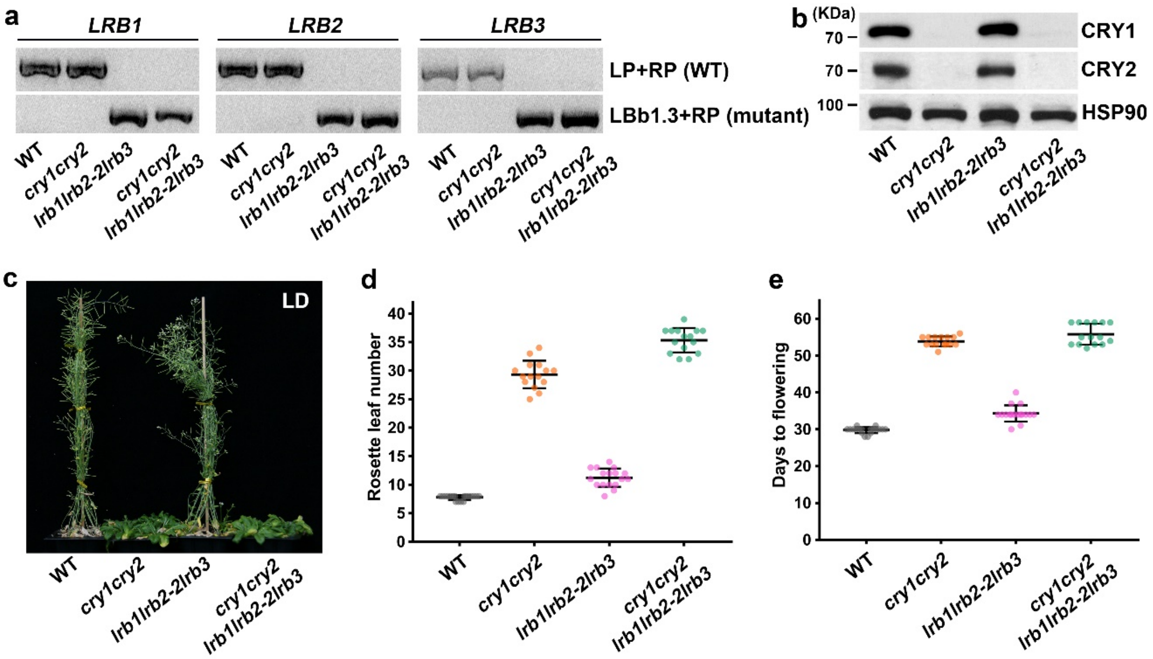

Supplementary Fig. 1 Flowering phenotype analysis of *cry1cry2lrb123* quintuple mutant plants. a PCR confirmation of T-DNA insertion at *LRB1*, *LRB2*, *LRB3* in *cry1cry2lrb123*. LP and RP primer pairs amplified the genomic DNA fragment of *LRB1*, *LRB2*, or *LRB3*. LBb1.3 and RP primers pair amplified the T-DNA insertion of *LRB1*, *LRB2*, or *LRB3*. **b** Immunoblots confirmation of the absence of CRY1 and CRY2 proteins in *cry1cry2lrb123*. Proteins isolated from indicated genotypes were analyzed with anti-CRY1 antibody, anti-CRY2 antibody or anti-HSP90 antibody. HSP90 is used as a loading control. **c** Representative images showing the flowering phenotypes of indicated plans grown in LD (16h light / 8h dark) for 54 days. **d-e** Quantitative analysis of rosette leaf number (**d**) or days to flowering (**e**) of indicated genotypes in LD condition. Data were shown as mean ± SD. The above experiments were repeated at least twice with similar results.

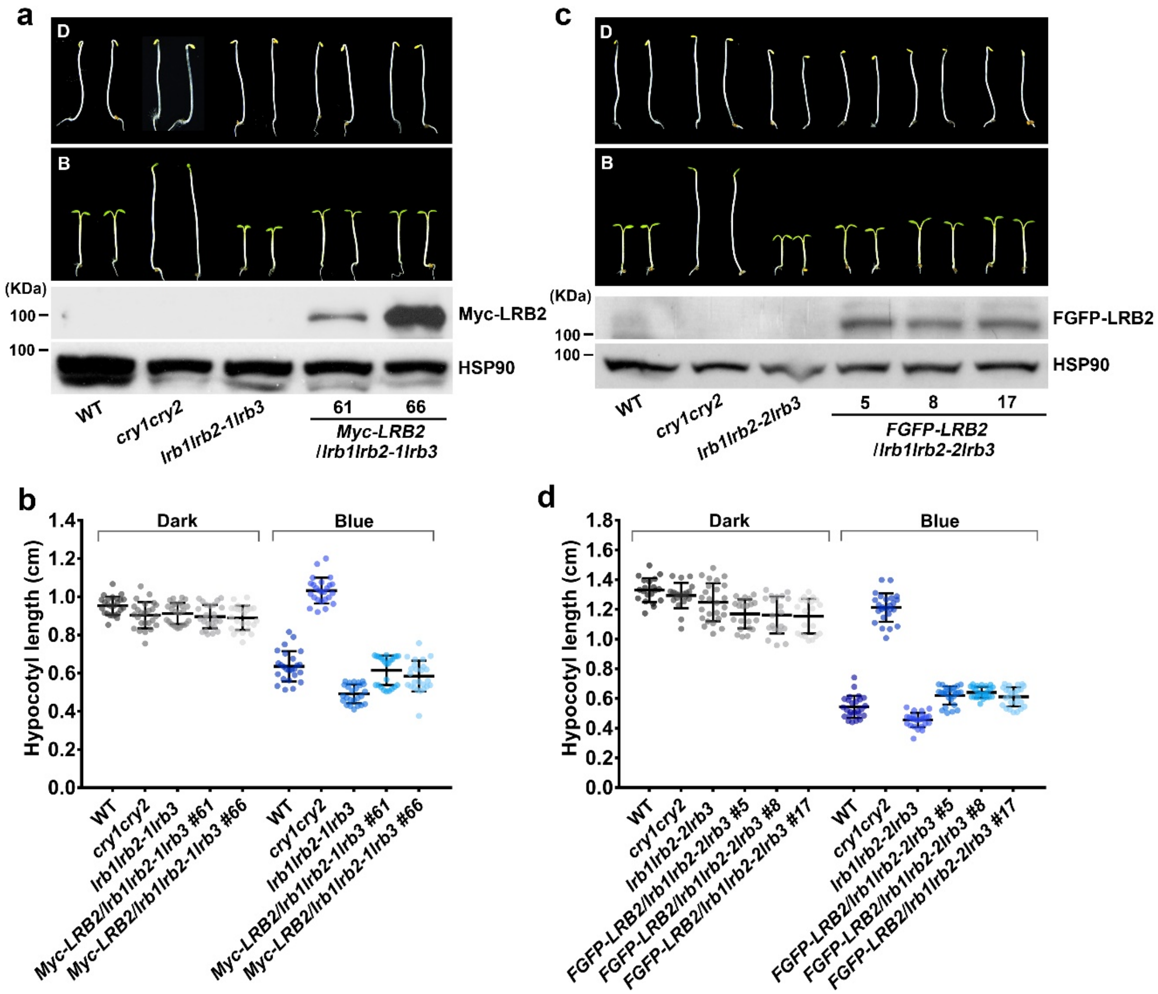

Supplementary Fig. 2 Rescue of the blue-light hypersensitivity phenotype of *lrb123* by LRB2. a, **c**, Seedlings were grown in darkness (D) or blue light (B, 5 μmol m^{-2} s^{-1}) for 6 days. Immunoblots showing the expression of Myc-LRB2 (**a**) and FGFP-LRB2 (**c**) in seedlings, were probed with anti-Myc, anti-Flag, or anti-HSP90 antibodies. HSP90 is used as a loading control. **b**, **d**, Measurements of hypocotyl length of seedlings shown in (**a**) or (**c**). Data were shown as mean ± SD. The above experiments were repeated at least three times with similar results.

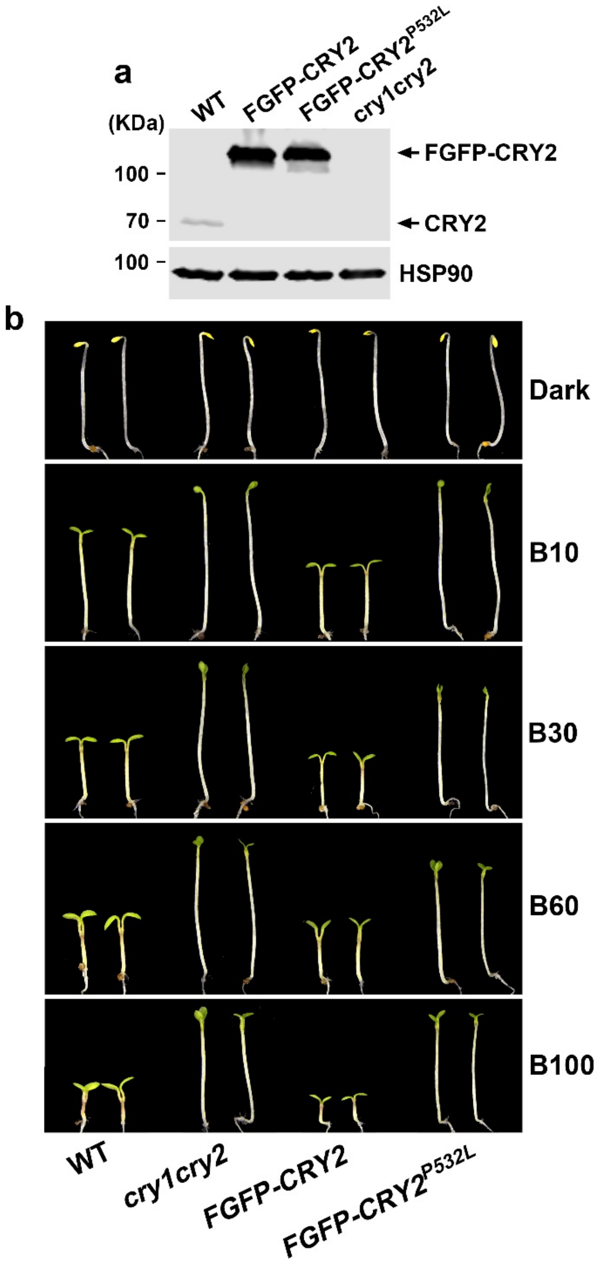

Supplementary Fig. 3 CRY2^{P532L} plant is insensitive to blue light. a, Proteins of 7-day-old seedlings were analyzed by immunoblots probed with anti-CRY2 antibody or anti-HSP90 antibody. HSP90 is used as a loading control. **b**, 6-day-old seedlings grown under different intensities of blue light (0, 10, 30, 60, 100 μmol m^{-2} s^{-1}). *FGFP-CRY2*, constitutively overexpressing FGFP-CRY2 in *cry1cry2rdr6*; *FGFP-CRY2^{P532L}*, constitutively overexpressing FGFP-CRY2^{P532L} in *cry1cry2rdr6*. The above experiments were repeated at least three times with similar results.

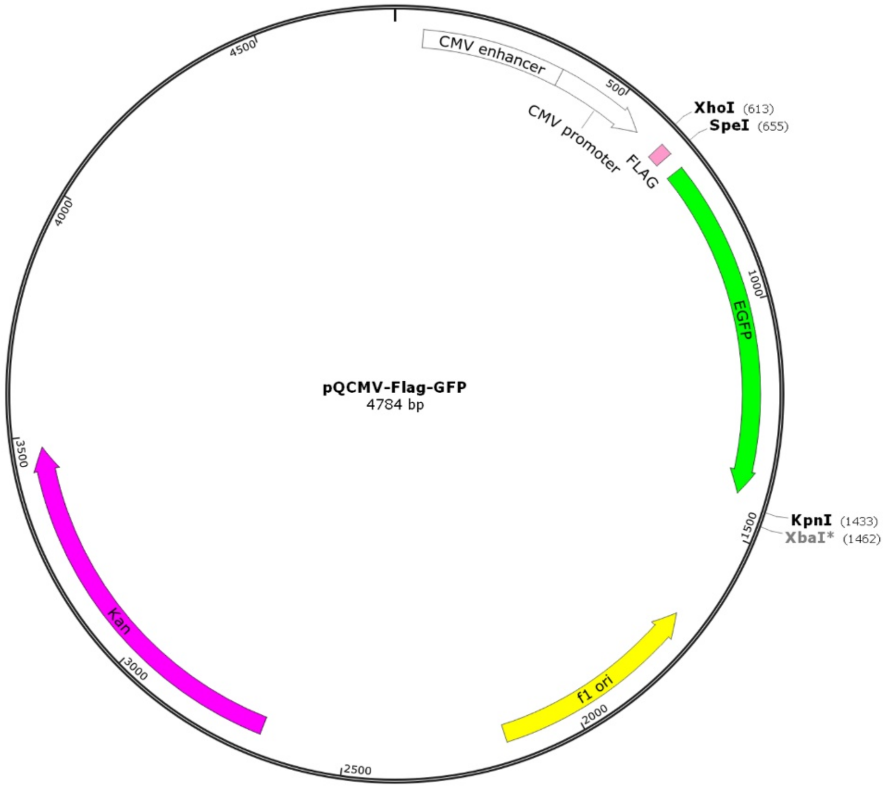

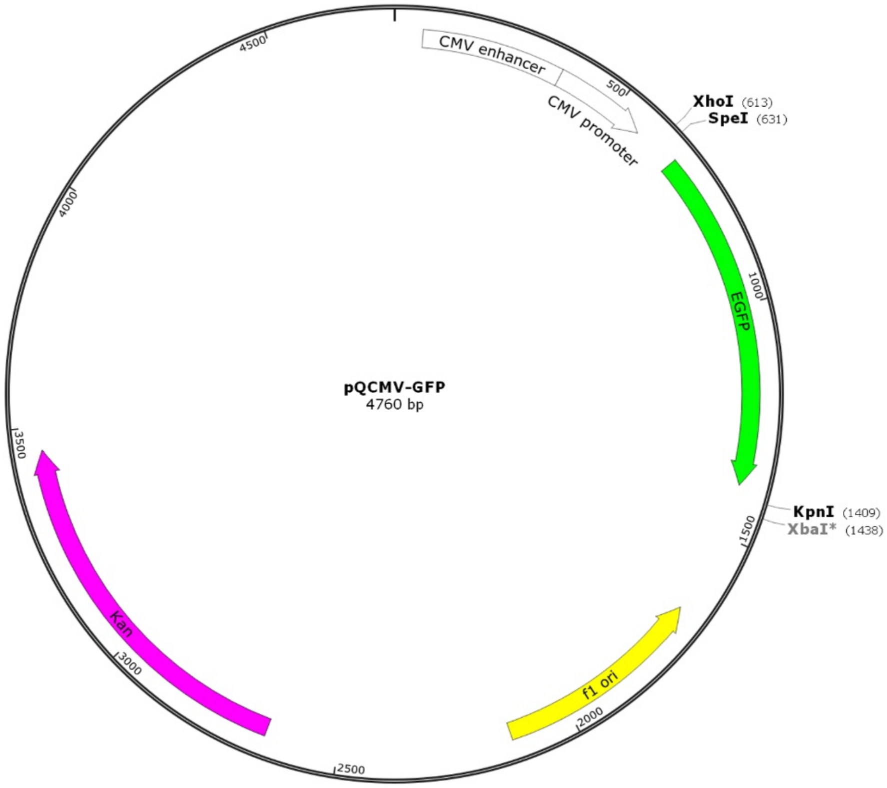

Supplementary Fig. 4 Maps of HEK293T vectors used in this study.

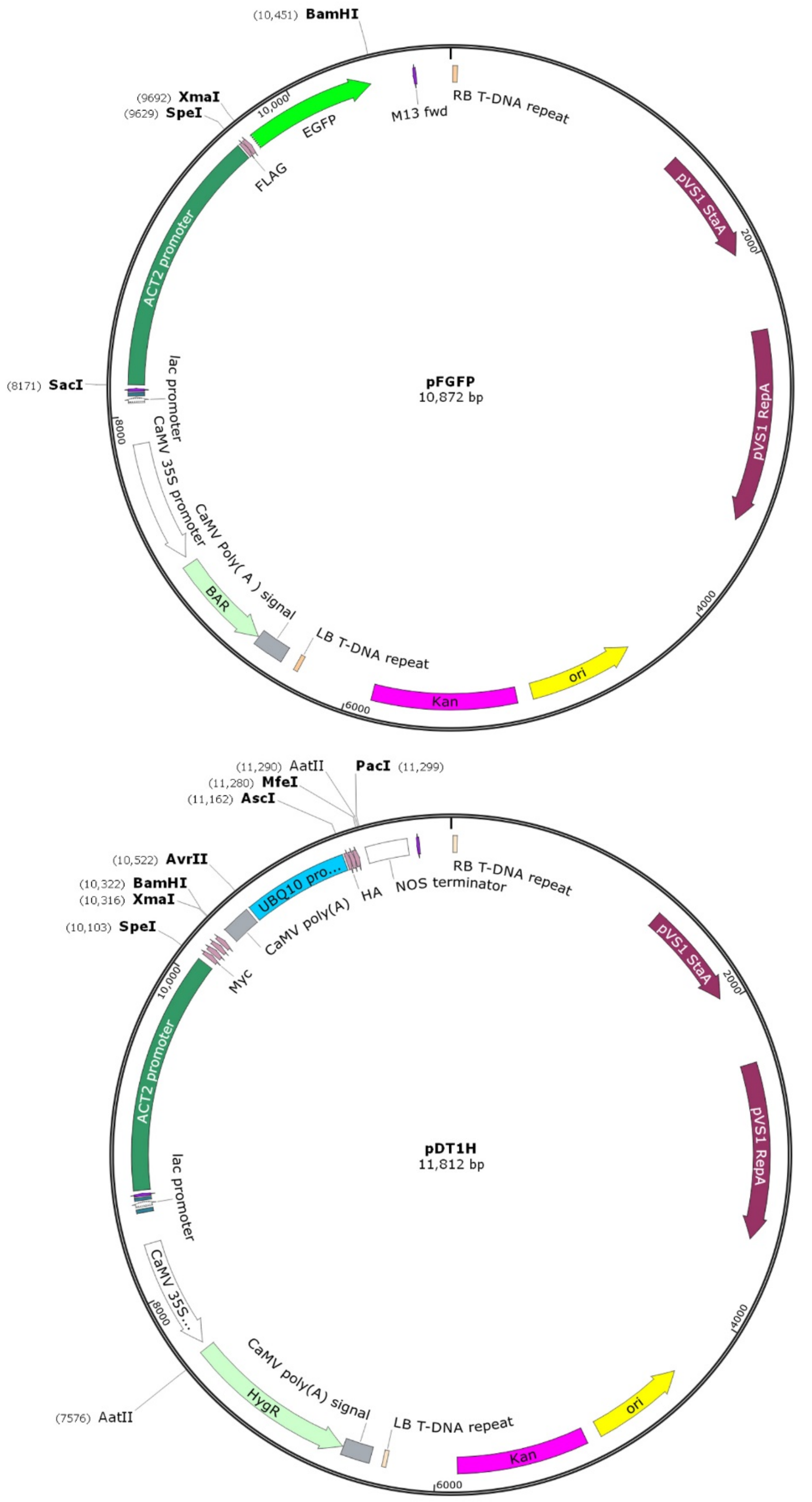

Supplementary Fig. 5 Maps of plant binary vectors used in this study.

Supplementary Table 1

Locus ID	Dark				Blue			
	Spectrum Count	NSAFe5	Sequence Count	Coverage (%)	Spectrum Count	NSAFe5	Sequence Count	Coverage (%)
CRY2	1423	23707.522	162	90.8	1371	14316.19	163	85.8
AT3G61600.1(LRB2)	0	0	0	0	10	113.91439	9	21
AT2G46260.1(LRB1)	0	0	0	0	3	34.174316	3	7

Supplementary Table 1 CRY2-IP mass-spectrometric results showing the blue light-dependent association of CRY2/LRB1 and CRY2/LRB2 in *Arabidopsis*. Dark-grown seedlings constitutively expressing *GFP-CRY2* were kept in the dark or irradiated with 30 μmol m^{-2} s^{-1} blue light for 10, 20 and 30 minutes before protein extraction. Proteins were immunoprecipitated by GFP-trap beads, precipitated, digested with trypsin and then subjected to mass-spectrometric analysis.

Supplementary Table 2. Primers used in this study

Primers for constructs used for expressing recombinant proteins in HEK293T Cells

Primer name	Primers
Flag-CRY2-F	CGACAAGGCTACTAGTATGAAGATGGACAAAAAGA CTATAGTT
Flag-CRY2-R	TAAGCGTGCTCAGCGGTACCTCATTTGCAACCATTT TTTCC
Flag-LRB1-F	CGACAAGGCTACTAGTATGAGAGGTTCCAATAACAC CGA
Flag-LRB1-R	TAAGCGTGCTCAGCGGTACCTCAGTGCAGGTCTGAG GAAC
Flag-LRB2-F	CGACAAGGCTACTAGTATGAGAGGTACTACTGAGAA TACGG
Flag-LRB2-R	TAAGCGTGCTCAGCGGTACCCTAAGGATCTGTAGAC CTTTTGATGG
Myc-CRY2-F	GGAGGACCTGGGATCCATGAAGATGGACAAAAAGAC TATAGTT
Myc-CRY2-R	TAGCAGGCCTGGATCCTCATTTGCAACCATTTTTCC
Myc-LRB2-F	GGAGGACCTGGGATCCATGAGAGGTACTACTGAGAA TACGG
Myc-LRB2-R	TAGCAGGCCTGGATCCCTAAGGATCTGTAGACCTTTT GATGG
Myc-COP1-F	GGAGGACCTGGGATCCATGGAAGAGATTTCGACGGA TCC
Myc-COP1-R	TAGCAGGCCTGGATCCTCACGCAGCGAGTACCAGAA CT
Myc-SPA1-F	GGAGGACCTGGGATCCATGCCTGTTATGGAAAGAGT AGC
Myc-SPA1-R	TAGCAGGCCTGGATCCTCAAACAAGTTTTAGTAGCTT CATG
HA-PPK1-F	CACCATGGCTACTAGTTACCCGTATGATGTTCCGGAT TACGCAGGTTACCCGTATGATGTTCCGGATTACGCAG GTATGCCTGAGCTGCGTAGC
HA-PPK1-R	TAAGCGTGCTCAGCGGTACCTCAAGATACAGTTCGGC CATAG

Primers for constructs used for BiFC assays

Primer name	Primers
pDONR-CRY2-F	GGGGACAAGTTTGTACAAAAAAGCAGGCTTCATGA AGATGGACAAAAAGACTATAGTT
pDONR-CRY2-R	GGGGACCACTTTGTACAAGAAAGCTGGGTCTTTGC AACCATTTTTTCCCA
pDONR-LRB1-F	GGGGACAAGTTTGTACAAAAAAGCAGGCTTCATGA GAGGTTCCAATAACACCGA
pDONR-LRB1-R	GGGGACCACTTTGTACAAGAAAGCTGGGTCGTGCA GGTCTGAGGAACG
pDONR-LRB2-F	GGGGACAAGTTTGTACAAAAAAGCAGGCTTCATGAG AGGTACTACTGAGAATACGG
pDONR-LRB2-R	GGGGACCACTTTGTACAAGAAAGCTGGGTCAGGATC TGTAGACCTTTTGATGG

Primers for constructs used for Split-LUC assays

Primer name	Primers
LRB1ΔBTB-nLUC-1F	CGGGGGACGAGCTCGGTACCATGAGAGGTT CCAATAACACCGAT
LRB1ΔBTB-nLUC-426R	ACCCCAGTTTGGCTCACTAGAT
LRB1ΔBTB-nLUC-637F	AACTGGGGTATTGATGCACCCGCTTTATTAG ATGTTCTTATG
LRB1ΔBTB-nLUC-1683R	ACGAGATCTGGTCGACGTGCAGGTCTGAGG AACGT
LRB2ΔBTB-nLUC-1F	CGGGGGACGAGCTCGGTACCATGAGAGGTA CTACTGAGAATACGGATC
LRB2ΔBTB-nLUC-432R	TCCCCAGTTTGGCTCACTAG
LRB2ΔBTB-nLUC-751F	GAGCCAAACTGGGGAGCCCTGCTCTATCTCG AGCT
LRB2ΔBTB-nLUC-1683R	ACGAGATCTGGTCGACAGGATCTGTAGACCT TTTGATGGTA
cLUC-CRY2-1F	ACGCGTCCCGGGGCGGTACCATGAAGATGG ACAAAAAGACTATAGTTTGGT
cLUC-CRY2-1839R	AGCTCTGCAGGTCGACTCATTTGCAACCATT TTTTCCCAAACTT

Primers for constructs used for plant transformation

Primer name	Primers
FGFP-CRY2-F	TCCAGCTCCAGGATCCATGAAGATGGACAAAAAGAC TATAGTT
FGFP-CRY2-R	GAGAAAGCTTGGATCCTCATTTGCAACCATTTTTTCC
FGFP-LRB2-F	TCCAGCTCCAGGATCCATGAGAGGTACTACTGAGAA TACGG
FGFP-LRB2-R	GAGAAAGCTTGGATCCCTAAGGATCTGTAGACCTTTT GATGG
Myc-LRB1-F	CACCCCCGGGGGATCCCCAGCTCCAGCTCCAATGAG AGGTTCCAATAACACCGA
Myc-LRB1-R	GAGAAAGCTTGGATCCTCAGTGCAGGTCTGAGGAACG T
Myc-LRB2-F	CACCCCCGGGGGATCCCCAGCTCCAGCTCCAATGAG AGGTACTACTGAGAATACGG
Myc-LRB2-R	GAGAAAGCTTGGATCCCTAAGGATCTGTAGACCTTTTG ATG
Myc-COP1-F	CACCCCCGGGGGATCCCCAGCTCCAGCTCCAATGGA AGAGATTTCGACGGATCC
Myc-COP1-R	GAGAAAGCTTGGATCCTCACGCAGCGAGTACCAGAACT
proLRB1-F	ATGATTACGAATTCGAGCTCTCTTAATTAGTCTCTGTTCT TCAAAACTTG
proLRB1-R	TGTAGTCCATACTAGTCCTCCTTTTCGAAAACCCTCTTC
proLRB2-F	ATGATTACGAATTCGAGCTCATAAAATGAGGCCCAGAT ACTTTTATTTG
proLRB2-R	TGTAGTCCATACTAGTCACCTTCCTTCAATTAATTTCCT AAGC

Primers for genotyping

Primer name	Primers
LBb1.3	ATTTTGCCGATTTCGGAAC
LRB1-LP	TGGCATTAACGAACCTCTTTG
LRB1-RP	GCGAGATGAACAAGAGCAAAC
LRB2-1-LP	CTGAACAGCTTTGGCCATTAG
LRB2-1-RP	TTCACTCGTCATGGTTCTTCC
LRB2-2-LP	CCAAAGCCAAAAGAGTAAGGG

LRB2-2-RP	TCAAGAGCACATGAGATGGTG
LRB3-LP	AAAAGCGCAAAAACCCTAAAC
LRB3-RP	CATTTGTTTTTGCTCCGACTC
COP1-1F	ATGGAAGAGATTTCGACGGATC
COP1-1327R	CTTCATAATCACTGCTTGCTATGTG
