# Supplementary material for: Regulation of Arabidopsis photoreceptor CRY2 by two distinct E3 ubiquitin ligases
Source: Nat Commun. 2021 Apr 12;12:2155. doi: 10.1038/s41467-021-22410-x (PMC8042123; doi:10.1038/s41467-021-22410-x)
Supplement: Supplementary file 3 — Reporting Summary [file 41467_2021_22410_MOESM3_ESM.pdf]

nature research

Corresponding author(s): Qin Wang
Last updated by author(s): Mar 8, 2021

Reporting Summary

Nature Research wishes to improve the reproducibility of the work that we publish. This form provides structure for consistency and transparency in reporting. For further information on Nature Research policies, see our Editorial Policies and the Editorial Policy Checklist.

Statistics

For all statistical analyses, confirm that the following items are present in the figure legend, table legend, main text, or Methods section.

n/a	Confirmed	
☐	☒	The exact sample size (n) for each experimental group/condition, given as a discrete number and unit of measurement
☐	☒	A statement on whether measurements were taken from distinct samples or whether the same sample was measured repeatedly
☒	☐	The statistical test(s) used AND whether they are one- or two-sided<br>*Only common tests should be described solely by name; describe more complex techniques in the Methods section.*
☒	☐	A description of all covariates tested
☒	☐	A description of any assumptions or corrections, such as tests of normality and adjustment for multiple comparisons
☐	☒	A full description of the statistical parameters including central tendency (e.g. means) or other basic estimates (e.g. regression coefficient) AND variation (e.g. standard deviation) or associated estimates of uncertainty (e.g. confidence intervals)
☒	☐	For null hypothesis testing, the test statistic (e.g. F, t, r) with confidence intervals, effect sizes, degrees of freedom and P value noted<br>*Give P values as exact values whenever suitable.*
☒	☐	For Bayesian analysis, information on the choice of priors and Markov chain Monte Carlo settings
☒	☐	For hierarchical and complex designs, identification of the appropriate level for tests and full reporting of outcomes
☒	☐	Estimates of effect sizes (e.g. Cohen's d, Pearson's r), indicating how they were calculated

Our web collection on statistics for biologists contains articles on many of the points above.

Software and code

Policy information about availability of computer code

Data collection	Image Studio Lite Ver 5.2 (Li-COR) was used for fluorescence immunoblot image acquisition.
Data analysis	Image Studio Lite Ver 5.2 (Li-COR) was used for fluorescence immunoblot quantification analysis; FIJI/ImageJ was used for ECL immunoblots and split-LUC quantification analysis; GraphPad Prism 7 was used for statistical analysis.

For manuscripts utilizing custom algorithms or software that are central to the research but not yet described in published literature, software must be made available to editors and reviewers. We strongly encourage code deposition in a community repository (e.g. GitHub). See the Nature Research guidelines for submitting code & software for further information.

Data

Policy information about availability of data

All manuscripts must include a data availability statement. This statement should provide the following information, where applicable:

- Accession codes, unique identifiers, or web links for publicly available datasets
- A list of figures that have associated raw data
- A description of any restrictions on data availability

The source data for Figs. 1-7, Supplementary Figs. 1-3 are provided with this paper as a Source Data file.
Other data and materials of this study are available from the corresponding author upon reasonable request.

Field-specific reporting

Please select the one below that is the best fit for your research. If you are not sure, read the appropriate sections before making your selection.

☒ Life sciences ☐ Behavioural & social sciences ☐ Ecological, evolutionary & environmental sciences

For a reference copy of the document with all sections, see nature.com/documents/nr-reporting-summary-flat.pdf

Life sciences study design

All studies must disclose on these points even when the disclosure is negative.

Sample size	No statistical methods were used to predetermine sample sizes. Required experimental sample sizes were estimated based on previous established protocols in the field. The sample sizes were adequate as the experimental results were reproducible.
Data exclusions	No data were excluded from the analysis.
Replication	All experimental findings were reproduced in several independent biological experiments (N) with multiple technical replicates. The number of repeats is indicated in the figure legends.
Randomization	Samples were randomly collected.
Blinding	Blinding was not possible as the authors who performed the experiments also analyzed the data.

Reporting for specific materials, systems and methods

We require information from authors about some types of materials, experimental systems and methods used in many studies. Here, indicate whether each material, system or method listed is relevant to your study. If you are not sure if a list item applies to your research, read the appropriate section before selecting a response.

Materials & experimental systems

n/a	Involved in the study	
☐	☒	Antibodies
☐	☒	Eukaryotic cell lines
☒	☐	Palaeontology and archaeology
☒	☐	Animals and other organisms
☒	☐	Human research participants
☒	☐	Clinical data
☒	☐	Dual use research of concern

Methods

n/a	Involved in the study	
☒	☐	ChIP-seq
☒	☐	Flow cytometry
☒	☐	MRI-based neuroimaging

Antibodies

Antibodies used	anti-CRY2 (homemade), anti-HSP82 (cat # AbM51099-31-PU, Beijing Protein Innovation), anti-Flag (cat # F1804, Sigma), anti-Myc (cat # 05-724, Millipore), anti-HA (cat # 12013819001, Roche), anti-Ubiquitin (cat # 14-6078-80, Thermofisher), anti-Rabbit-HRP (cat # 31460, ThermoFisher), anti-mouse-HRP (cat # 31430, ThermoFisher), Donkey anti-rabbit 790 (cat # A11374, ThermoFisher), Donkey anti-mouse 680 (cat # A10038, ThermoFisher).
Validation	Validation of anti-CRY2 antibodies was described in the reference: Science. 1998. 279:1360-1363;<br>validation of all the other commercial antibodies could be found on the manufacturers' websites.

Eukaryotic cell lines

Policy information about cell lines

Cell line source(s)	Human embryo kidney cells (HEK293T) (ATCC, CRL-11268)
Authentication	The HEK293T cell line used in this study is not authenticated.
Mycoplasma contamination	The HEK293T cell line used in this study was not specifically tested for Mycoplasma contamination. However, no abnormalities of cultured cells have been observed, such as slowed cell growth and interfered cell attachment.
Commonly misidentified lines<br>(See ICLAC register)	*Name any commonly misidentified cell lines used in the study and provide a rationale for their use.*
